# Serum GGT activity and hsCRP level in patients with type 2 diabetes mellitus with good and poor glycemic control: An evidence linking oxidative stress, inflammation and glycemic control

**DOI:** 10.1186/2251-6581-12-56

**Published:** 2013-12-20

**Authors:** Mukesh G Gohel, Anusha N Chacko

**Affiliations:** 1 Department of Biochemistry, B. J. Medical College & Civil Hospital, Ahmedabad, India; 2 Department of Biochemistry, GDERS Dental College, Siddhapur, India

**Keywords:** Glycemic Control, Diabetes Mellitus Patient, Poor Glycemic Control, hsCRP Level, Good Glycemic Control

## Abstract

**Background:**

Diabetes is undoubtedly one of the most challenging health problems in 21^st^ century. Understanding the pathogenesis and preventing long term complications have been major goals of research in diabetes mellitus (DM). Research in the past few years has linked oxidative stress and inflammation to beta cell dysfunction. Aim of this study is to evaluate serum gamma-glutamyl transferase (GGT) activity (marker of oxidative stress) and high sensitivity C reactive protein (hsCRP) level (an inflammatory marker) in type 2 DM subjects with good and poor glycemic control. Further, we investigated correlation between serum GGT and hsCRP level with glycemic control (FBS, PP_2_BS, HbA_1_c) in subjects.

**Methods:**

A cross sectional study consists of 150 patients out of them 50 patients having type 2 DM with good control (Group II), 50 patients with type 2 DM with poor control (Group III) and 50 normal healthy control (Group I) were selected. Serum GGT, serum hsCRP, FBS, PP_2_BS, HbA_1_c, and other biochemical investigations include serum liver enzymes and lipids were measured.

**Results:**

Mean serum GGT and hsCRP concentration were statistically significantly higher in group III patients compared to group I and group II subjects as well as increased in group II compared to group I (p < 0.001). Further significant positive correlation was observed between GGT and hsCRP concentration as well as both with HbA_1_c, FBS, and PP_2_BS.

**Conclusions:**

Oxidative stress and inflammation appears to be a key component and also associated with poor glycemic control and further pathogenesis of diabetes and its complications. All our finding suggesting a link between oxidative stress, inflammation and glycemic control in patient with type 2 diabetes mellitus.

**Electronic supplementary material:**

The online version of this article (doi:10.1186/2251-6581-12-56) contains supplementary material, which is available to authorized users.

## Background

Diabetes mellitus (DM) comprises a group of common metabolic disorders that share common phenotype of hyperglycemia[1]. Hyperglycemia not only defines the disease but is the cause of its most characteristic symptoms and long-term complications. Understanding the pathogenesis and preventing long-term complications have been major goals of research in diabetes mellitus. Diabetes is undoubtedly one of the most challenging health problems in 21^st^ century[2]. Research in the past few years has linked oxidative stress and inflammation to β-cell dysfunction resulting from chronic exposure to hyperglycemia. A growing body of data reinforces the concept that inflammation plays an important role in the pathogenesis of type 2 DM and links DM with concomitant conditions with inflammatory components[3].

Gamma-glutamyl transferase (GGT, E.C.2.3.2.2) is a cell-surface protein contributing to the extracellular catabolism of glutathione (GSH). The enzyme is produced in many tissues, but most GGT in serum is derived from the liver[4]. GGT has a pivotal role in the maintenance of intracellular antioxidant defences through its mediation of extracellular glutathione (GSH) transport into most types of cells[5]. Oxidative stress is associated with a number of pathological conditions, such as inflammation, carcinogenesis, aging, atherosclerosis, and reperfusion injury[6]. Oxidative stress can also play a role in the cause and pathophysiology of diabetes. Most studies focus on the role of oxidative stress in developing cardiovascular complications in diabetic patients,[7]. Moreover, elevated GGT is strongly associated with obesity and excess deposition of fat in the liver, termed non-alcoholic fatty liver disease, which is thought to cause hepatic insulin resistance and to contribute to the development of systemic insulin resistance and hyperinsulinemia[8]. Thus, GGT might reflect metabolic alterations and could serve as a marker of the insulin resistance syndrome. Other studies suggested that GGT plays an important role in antioxidant systems[5, 9].

High sensitivity C-reactive protein (hsCRP) is a C-reactive protein measured by a highly sensitive assay. CRP represents the classical acute-phase protein produced in the liver in response to inflammatory stimuli, and plasma levels of hsCRP provide a sensitive marker of increased inflammatory activity in the arterial wall[10]. Chronic, systemic subclinical inflammation has also been identified as a driving force for insulin resistance, metabolic syndrome, and type 2 DM. Some related metabolic disorders include abdominal adiposity, hypertension, endothelial dysfunction, and glucose intolerance, which often occur in a cluster. Insulin resistance correlates closely with the risk of cardiovascular diseases (CVD), explaining some of the excess morbidity and mortality in type 2 DM patients[11, 12].

Because the development of complications is linked to the accumulation of glycation adducts in tissue proteins. The core of the issue is glycemic control. Optimal monitoring of glycemic control involves plasma glucose measurements (fasting and postprandial blood sugar) and measurement of glycated hemoglobin (HbA_1_c). These measurements are complementary: the patient’s glucose measurements provide a picture of short-term glycemic control, whereas HbA_1_c reflects average glycemic control over the previous 3 months[1].

Since, oxidative stress and inflammation appears to be a key component of many reactions associated with poor glycemic control and further pathogenesis of diabetes and its complications; we found it interesting to study serum GGT activity (marker of oxidative stress) and hsCRP level (an inflammatory marker) in diabetic subjects. Further, we investigated correlation between serum GGT and hsCRP with glycemic control in subjects.

## Methods

### Study design and subjects

This study was a hospital based cross sectional study conducted at shree sayajirao general hospital and medical college, Vadodara (India) between May 2009 to June 2010. A cross sectional study consists of 150 subjects out of them 50 patients having type 2 DM with good glycemic control (Group II), 50 patients with type 2 DM with poor glycemic control (Group III) and 50 normal healthy control (Group I) were selected. Subjects were recruited according to simple random sampling method meeting the selection criteria.

### Selection criteria

#### Inclusion criteria

The subjects selected for study were grouped as follows:

##### Group I – Control group (n=50)

This group consisted of age and sex matched healthy subjects. They were free from any ailment which could affect the parameters under study. They were not on any medication. They were taken from general population.

##### Group II – Type 2 DM patients with good glycemic control (n=50)

This group consisted of patients with type 2 DM with duration less than 8 years, HbA_1_c level less than 7%. They were on life style modifications and oral hypoglycemic drugs and free from clinical evidence of any complication of diabetes mellitus.

##### Group III – Type 2 DM patients with poor glycemic control (n=50)

This group consisted of patients with type 2 DM with duration more than 8 years, HbA_1_c level more than 7%. They were on life style modifications, oral hypoglycemic drugs, insulin or combination of all three and associated with one or more microvascular or macrovascular complication of diabetes mellitus for e.g. diabetic retinopathy, heart disease, diabetic neuropathy.

#### Exclusion criteria

The patients with type 1 diabetes mellitus, high (>30 g/d) alcohol consumption, with known liver or gastrointestinal diseases, with liver enzyme concentrations higher than three times the upper limit, on corticosteroids, methotrexate, amiodarone, tamoxifen or other hepatotoxic drugs, any chronic infection like tuberculosis, sarcoidosis etc.,, hemolytic anaemia, hemoglobin variants were excluded from this study.

### Ethical considerations

The objectives of study were explained to all eligible subjects for this study. Informed written consent of all subjects included in the study was obtained for involvement in study groups and for venipuncture. Emphasis was given that participation in this study was voluntary.

### Questionnaire and data collection

A questionnaire was specifically designed to obtain information which helps to select individuals according to the selection criteria of the study. The questions also focused on baseline information on socio-demographic variables (age, sex) and background characteristics of diabetes (duration and type of diabetes mellitus, mode of anti-diabetic therapy, any complication) during a standardized interview. In addition, all participants underwent an extensive standardized medical examination including the collection of blood sample.

### Blood sample collection

A 5 ml of venous blood was drawn from each volunteer using a disposable vacutainer system in fasting condition (Plain, EDTA and Fluoride). Post prandial (2 hour) sample collected in fluoride vacutainer for PP_2_BS estimation. Serum or plasma separated within half an hour and stored at 2-8°C temperature till analysis was done.

### Analysis of sample

Fasting and post prandial (2 hour) blood sugar (FBS & PP_2_BS) estimated by glucose oxidase-peroxidase (GOD-POD) enzymatic end point method (Kit: Quantitative determination blood sugar by glucose oxidase peroxidase method mfg by Spinreact). Glycated hemoglobin (HbA_1_c) concentration was measured by immuno turbidimetric method (Kit: Quantitative determination of glycated hemoglobin (HbA_1_c) in human blood by latex turbidimetry mfg by Spinreact). Serum GGT activity was determined by carboxy substrate kinetic method. (Kit: Quantitative determination of GGT by carboxy substrate method mfg by Coral Crest biosystems). Serum hsCRP level is measured by immuno turbidimetric method (Kit: Quantitative determination of hsCRP in human blood by latex turbidimetry assay mfg by Spinreact). All other biochemical investigation includes serum liver enzymes, lipids, and other biochemical blood measurements were determined using standard laboratory procedures on fully automatic analyzer I.S.E. srl MIURA. Hemogram and urine examination were done in pathology laboratory. Fundoscopy and electrocardiogram were done in respective department.

### Statistical analysis

The data collected during the current study were recorded and analysed statistically to determine the significance of different parameters by using GraphPad InStat statistical software. Results are expressed as mean ± SD. The values between groups are compared using one way ANOVA test. *P* value of < 0.05 was considered statistically significant. Pearson linear correlation was used to study correlation between parameters.

## Results

**Figure 1:**
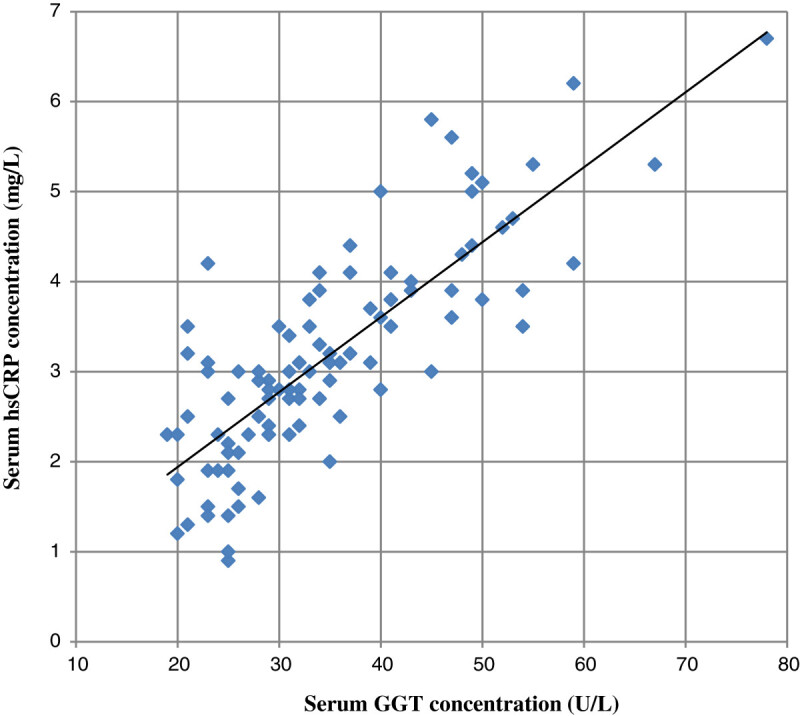
**Showing correlation between serum GGT and hsCRP concentration in patients.**

**Figure 2:**
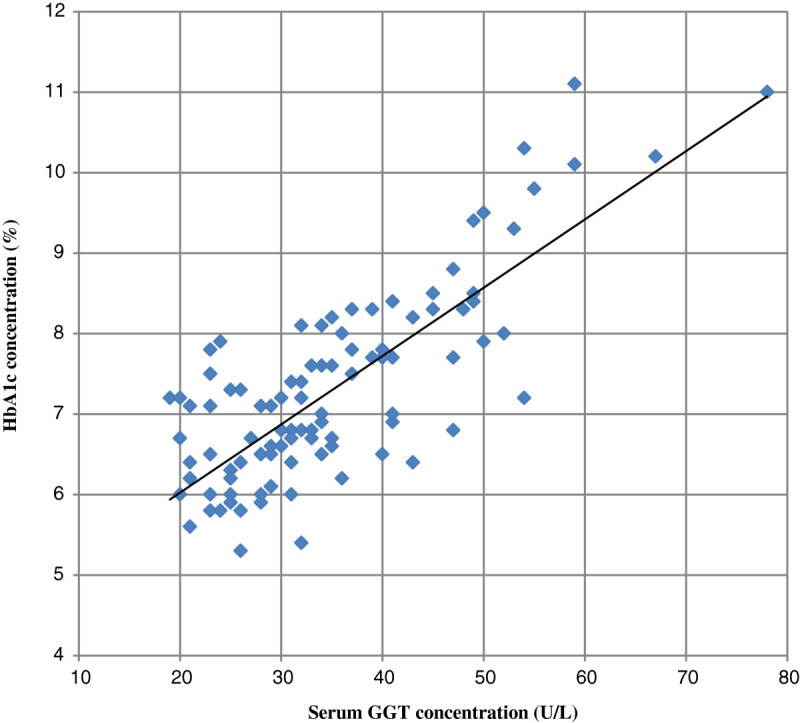
**Showing correlation between serum GGT and HbA**_**1**_ **c concentration in patients.**

Our study shows mean serum GGT concentration in group III was significantly higher compared to control (group I) and group II cases (p values < 0.001). Also serum GGT concentration in group II was significantly higher compared to group I (p values < 0.05). Further mean hsCRP levels in group III and group II were significantly higher compared to group I (p values < 0.001). Also hsCRP level in group III was higher than group II (p values < 0.001) (Table 1). We have found a statistically significant positive linear relationship between serum GGT and serum hsCRP concentration and with glycemic control (HbA_1_c, FBS, and PP_2_BS). Also a significant positive association was observed between serum hsCRP level with glycemic control (HbA_1_c, FBS, and PP_2_BS) (Table 2; Figures 1,2 and3).

**Table 1:** **Values of Serum GGT and hsCRP concentration between study groups**

Study Groups	GGT (U/L)	hsCRP (mg/L)
Group I	24.78 ± 5.8	1.4 ± 0.4
Group II	30.06 ± 7.02	2.50 ± 0.78
t value	2.93	6.8
p values	< 0.05	< 0.001
Group II	30.06 ± 7.02	2.50 ± 0.78
Group III	39.5 ± 12.68	3.84 ± 1.09
t value	5.23	8.28
p values	< 0.001	< 0.001
Group I	24.78 ± 5.8	1.4 ± 0.4
Group III	39.5 ± 12.68	3.84 ± 1.09
t value	8.16	15.09
p values	< 0.001	< 0.001

**Table 2:** **Pearson’s correlation analysis between serum GGT, hsCRP and glycemic control**

	Correlation coefficient r value	Two tailed p value
Serum GGT with hsCRP	0.80	< 0.0001
Serum GGT with HbA_1_c	0.79	< 0.0001
Serum GGT with FBS	0.54	< 0.0001
Serum GGT with PP_2_BS	0.53	< 0.0001
Serum hsCRP with HbA_1_c	0.78	< 0.0001
Serum hsCRP with FBS	0.56	< 0.0001
Serum hsCRP with PP_2_BS	0.63	< 0.0001

**Figure 3:**
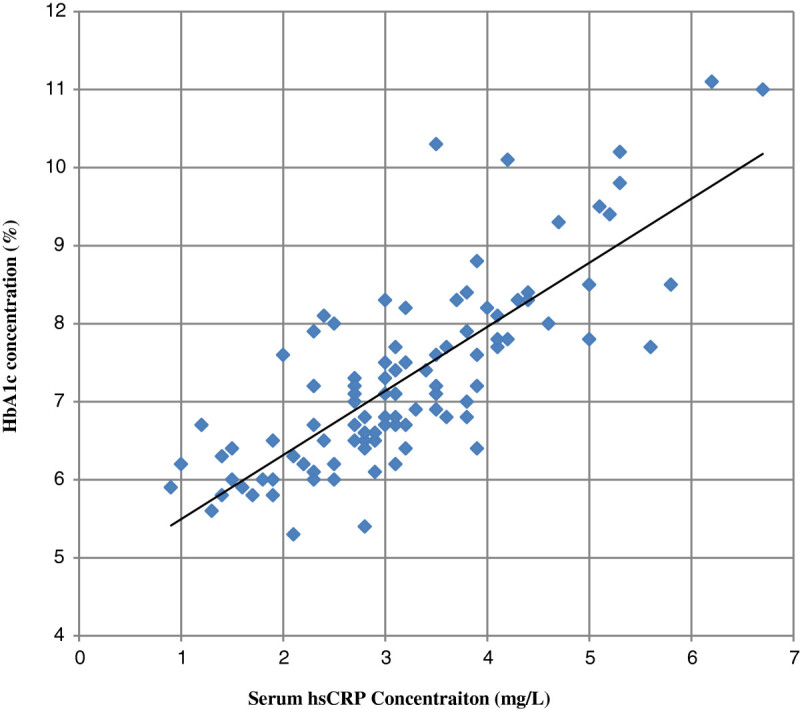
**Showing correlation between serum hsCRP and HbA**_**1**_ **c concentration in patients.**

Baseline characteristics of control and subjects as well other biochemical parameter are given in Table 3. Baseline characteristics like age, sex, blood pressure, BMI were not differing between groups. We found significantly increased serum alanine transaminase (ALT) and aspartate transaminase (AST) concentration in group III compared to group I and group II (p values < 0.001). But serum alkaline phosphatase (ALP) concentration is not increased between groups (p value is 0.62 which considered not significant).

**Table 3:** **Comparison of baseline characteristics and other biochemical parameters between study groups**

	Group I	Group II	Group III
Number of subjects	50	50	50
Sex (M/F) %	53/47	51/49	55/45
Age (In Years)	56	55	59
Duration of diabetes (In years)	-	5.11	12.28
Height (cm)	155.94 ± 8.029	154.3 ± 4.22	157.4 ± 4.41
Weight (kg)	57.96 ± 4.535	63.6 ± 5.67	66.6 ± 5.78
BMI	24.16 ± 5.1359	26.77 ± 2.877	26.86 ± 2.48
Serum GGT concentration (U/L)	24.78 ± 5.8	30.06 ± 7.02	39.5 ± 12.68
Serum hsCRP concentration (mg/L)	1.4 ± 0.4	2.50 ± 0.78	3.84 ± 1.09
HbA_1_c (%)	5.48 ± 0.48	6.36 ± 0.43	8.19 ± 1.03
FBS (mg/dl)	90.68 ± 14.58	125.64 ± 27.08	194.26 ± 56.6
PP_2_BS (mg/dl)	113.18 ± 14.18	158.54 ± 23.85	274.08 ± 63.00
Total Cholesterol (mg/dl)	147.7 ± 25.9	193.32 ± 51.5	201.76 ± 58.17
Triglycerides total (mg/dl)	103.36 ± 23.2	130.98 ± 32.0	140.56 ± 65.2
ALT (U/L)	18.52 ± 6.0	21.46 ± 20.7	27.8 ± 5.66
AST (U/L)	19.12 ± 5.3	21.3 ± 5.6	25.9 ± 6.1
ALP (U/L)	140.36 ± 34.6	147.4 ± 30.2	144.2 ± 43.1

## Discussion

Our study shows statistical significantly increased concentration of GGT and hsCRP in serum in patient with type 2 DM with poor glycemic control compared to healthy persons as well as subjects having type 2 DM with good glycemic control. Also we found a significant positive linear relationship between GGT and hsCRP concentration as well as both with HbA1C, FBS, and PP_2_BS. These findings suggest a link between oxidative stress (indicated by increased serum GGT concentration), inflammation (raised hsCRP concentration) and glycemic control in patients with type 2 DM and related complications. Also at levels of GGT and hsCRP considered well within the normal range, there was a substantial and significant increased concentration in patients with type 2 DM with good glycemic control compared to healthy subjects. This suggests a role of oxidative stress and chronic low grade inflammation in pathogenesis of type 2 diabetes patients.

Several possible mechanisms which explain increased serum GGT activity and hsCRP level in patients with type 2 DM with good and poor control and its correlation with glycemic control.

Elevation of serum GGT could be the expression of an excess deposition of fat in the liver, termed non-alcoholic fatty liver disease. Fatty liver is thought to cause hepatic insulin resistance and to contribute to the development of systemic insulin resistance and hyperinsulinemia. Thus, GGT could serve as a marker of the insulin resistance syndrome in the pathogenesis of diabetes[8, 13].

There is now growing evidence to suggest that GGT is not only a marker of fatty liver but also a marker of oxidative stress. Experimental studies have reported that GGT has a central role in the maintenance of intracellular antioxidant defences through its mediation of extracellular glutathione transport into most types of cells[5]. It is an ectoenzyme normally present at the outer side of the cell membrane that has the primary function of maintaining intracellular concentrations of glutathione (GSH), a critical antioxidant defence for the cell. Increases in GGT activity can be a response to oxidative stress, facilitating increased transport of GSH precursors into cells. In addition, GGT is leaked into the serum possibly as a result of normal cell turnover and cellular stresses. Several mechanisms for GGT leakage are possible and include increases in oxidative stress, proteolysis, glycosylation, GGT synthesis and endothelial cell damage[14]. Thus, increased serum concentrations of GGT could identify people with a low but persistent increase of oxidative and other cellular stresses.

Oxidative stress is currently suggested as mechanism underlying diabetes and diabetic complications. In recent years, much attention has been focused on the role of oxidative stress, and it has been reported that oxidative stress may constitute the key and common event in the pathogenesis of secondary diabetic complications. Implication of oxidative stress in the pathogenesis of diabetes is suggested, not only by oxygen free-radical generation, but also due to nonenzymatic protein glycosylation, auto-oxidation of glucose, impaired glutathione metabolism, alteration in antioxidant enzymes, lipid peroxides formation and decreased ascorbic acid levels. In addition to GSH, there are other defence mechanisms against free radicals like enzymes superoxide dismutase (SOD), glutathione peroxidase (GPx) and catalase (CAT) whose activities contribute to eliminate superoxide, hydrogen peroxide and hydroxyl radicals[15]. Raised GGT concentrations could be a marker of oxidative stress, which might also play a role in the cause and development of diabetes and its complications[5, 9].

Various studies suggested that elevated serum GGT could be the expression of subclinical inflammation which also contributes to the development of type 2 DM[16, 17]. As serum GGT is highly associated with WBC count and some features of low-graded inflammation, so elevated GGT could be the expression of subclinical inflammation or an insulin-resistant state, which would represent the underlying mechanism[17, 18]. It is therefore suggested that measurements of other inflammatory markers including C-reactive protein by a validated high-sensitivity assay be added in an attempt to substantiate this hypothesis.

There are various studies which support our results. R Sharma et al. shows rise in levels of hsCRP and GGT in diabetic subjects and their significant association which might be a result of inflammation and oxidative stress in diabetes mellitus[19]. Dilshad Ahmed Khan et al. studied diabetic patients had significantly elevated median of HbA_1_c, hsCRP, total cholesterol, nitrate and GGT as compared to controls. HbA_1_c showed a positive correlation with hsCRP, total cholesterol, nitrate and GGT. Oxidative stress and inflammatory markers should be used in addition to HbA_1_c for assessment of increased cardiac risk in uncontrolled diabetic patients because of accelerated atherosclerosis due to free radical injury[20]. Sarinnapakorn V et al. found that hsCRP levels correlated with HbA_1_c levels. Mean HbA_1_c levels were significantly higher in patients who had hsCRP levels of 1 mg/L or more. Other factors such as age, blood pressure, BMI, LDL cholesterol, serum creatinine were not correlated with hsCRP level[21]. Also Bahceci M et al. compare serum hsCRP levels in type 2 diabetic men without coronary heart diseases (CHD), non-diabetic CHD patients and type 2 DM patients with CHD and shows type 2 DM men without CHD had similar CRP levels with non-diabetic CHD patients, whereas CRP levels of type 2 DM men with CHD were higher than non-diabetic men with CHD. Because of a positive correlation between serum hsCRP and HbA_1_c, fasting insulin and HOMA-IR, inflammation, insulin resistance and hyperglycemia jointly contribute to the cardiovascular risk in type 2 DM men[22].

Other lines of evidence support a relationship between elevated serum GGT and poor glycemic control and metabolic syndrome are also found. Higher GGT levels are accompanied by more insulin resistance and greater risk for developing type 2 DM and poor glycemic control[23–27]. The strong association of serum GGT activity with some diabetes related metabolic disorders, such as atherogenic dyslipidemia and poor glycemic control, may be explained by underlying, not mutually exclusive, biological mechanisms such as fatty liver, insulin resistance, and enhanced oxidative stress[23, 28–32]. It is possible that the occurrence of GGT-mediated redox reactions plays a direct role in the pathogenesis of atherogenic dyslipidemia and poor glycemic control, independently of the presence of fatty liver, possibly through the induction of chronic inflammation and insulin resistance[33]. Supporting a role of serum GGT in the inflammation and oxidative stress, serum GGT level predicted future levels of inflammation and oxidative stress markers, such as fibrinogen, uric acid, CRP, and F2-isoprostanes, in a dose–response manner[34]. Several studies demonstrate that hsCRP remained a significant predictor of diabetes risk even after adjusting with body mass index, family history of diabetes mellitus, smoking and other factors.

In general, serum GGT concentration is closely related with other enzymes more specific to the liver, serum ALT or AST concentration, so we did parallel analyses with ALT and AST to further explore the possible role of liver damage in the association of GGT with diabetes. Within their normal ranges, ALT and AST showed significant increase in type 2 diabetes patients with poor control (p value <0.001). Moreover, markers of hepatic fat content, such as serum GGT activity and other liver enzymes, have been shown in large prospective studies to predict the incidence of type 2 diabetes, insulin resistance, and cardiovascular disease independently of obesity[23, 28, 35, 36].

Our results also suggest that liver enzymes are closely associated with the risk of metabolic syndrome and type 2 diabetes and that among these enzymes serum GGT is the most powerful risk indicator for developing the metabolic syndrome and type 2 diabetes. Another possible pathophysiological mechanism is that elevated liver enzymes may reflect inflammation, which impairs insulin signalling both in the liver and systemically[27, 37, 38]. In this population, mean WBC count increased with an increase in serum GGT. WBCs, a major component of the inflammatory process, are activated by cytokines, especially interleukin-6 and interleukin-8[39]. Elevated GGT could reflect subclinical inflammation, which would represent the underlying mechanism. Serum GGT level may be a simple and reliable marker of visceral and hepatic fat and, by inference, of hepatic insulin resistance. These findings suggest that a raised serum GGT level is an independent risk factor for NIDDM. The elevated GGT concentration should be added to the list of risk factors that are common to NIDDM and cardiovascular disease[13].

Our study had several limitations. First, it is cross sectional study with no causality effect to report. Also serum GGT and hsCRP during follow-up was not included in the analysis. Further, we could not include several confounding variables in this study, such as fasting insulin concentration. Therefore, fasting insulin concentration should be included in future studies.

Despite these potential limitations, our findings, which were obtained from a cross sectional study shows that serum GGT activity and hsCRP level is significantly increased in patients with type 2 diabetes mellitus compared to healthy control. Both are further increased in diabetic patients with complications and poor glycemic control. Also there is a significant positive correlation between serum GGT activity and hsCRP. Both are also independently positively correlated with HbA_1_c, FBS and PP_2_BS (short and long term glycemic control). So far, the underlying pathophysiological mechanisms are not entirely clear. It seems that insulin resistance, oxidative stress and chronic low grade systemic inflammation may be involved. All these finding suggesting a link between oxidative stress, inflammation and glycemic control in patient with type 2 diabetes mellitus. Further studies are needed to investigate the biological mechanisms underlying this association.

## Conclusions

In conclusion, the present study suggests that serum GGT and hsCRP concentration is significantly increased in type 2 diabetes mellitus. Both are further increased in diabetic patients with complications and poor glycemic control. There is a significant positive correlation between serum GGT activity and hsCRP. Serum GGT level and hsCRP concentration was independently and positively correlated with FBS, PP2BS and HbA1c (markers of glycemic control). All these finding suggesting a link between oxidative stress, inflammation and glycemic control in patient with type 2 diabetes mellitus.

## Authors’ original submitted files for images

Below are the links to the authors’ original submitted files for images. Authors’ original file for figure 1 Authors’ original file for figure 2 Authors’ original file for figure 3
