# Supplementary figures and images for: Serum GGT activity and hsCRP level in patients with type 2 diabetes mellitus with good and poor glycemic control: An evidence linking oxidative stress, inflammation and glycemic control
Source: J Diabetes Metab Disord. 2013 Dec 20;12:56. doi: 10.1186/2251-6581-12-56 (PMC7962520; doi:10.1186/2251-6581-12-56)

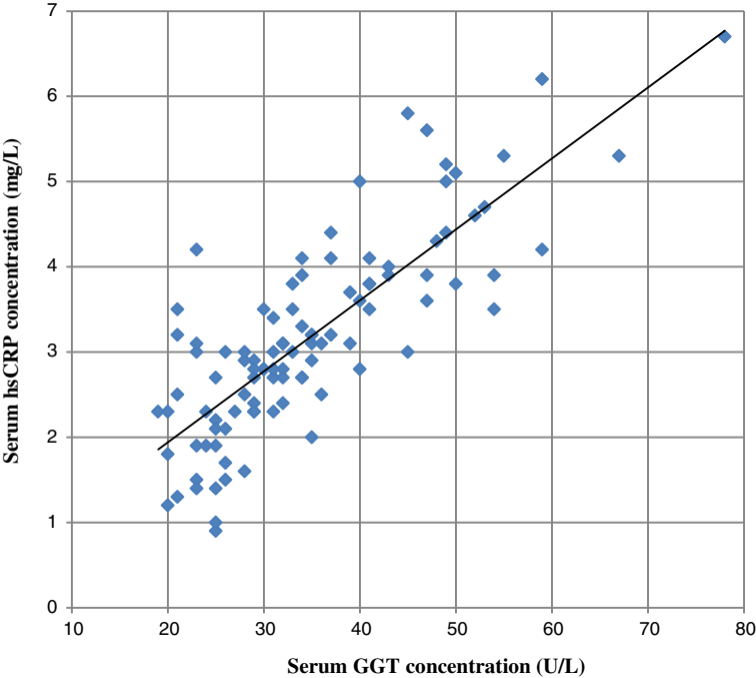

Supplement: Supplementary file 1 — Authors’ original file for figure 1 [file 40200_2013_188_MOESM1_ESM.pdf]

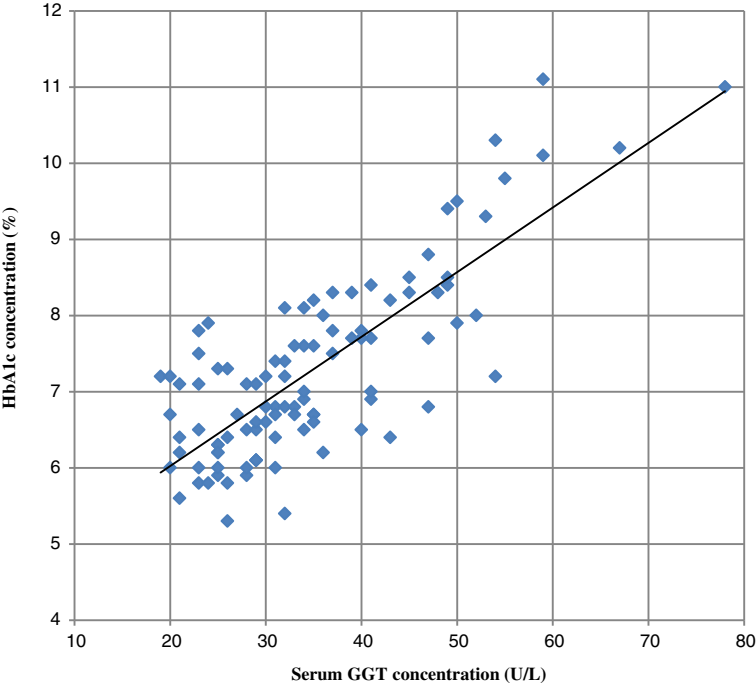

Supplement: Supplementary file 2 — Authors’ original file for figure 2 [file 40200_2013_188_MOESM2_ESM.pdf]

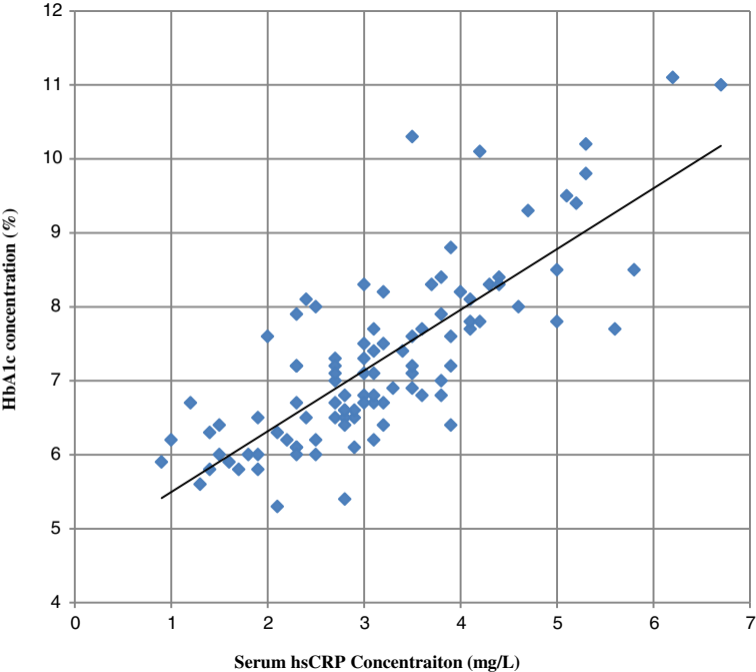

Supplement: Supplementary file 3 — Authors’ original file for figure 3 [file 40200_2013_188_MOESM3_ESM.pdf]
